# Supplementary material for: Probabilistic Optically-Selective Single-molecule Imaging Based Localization Encoded (POSSIBLE) microscopy for ultra-superresolution imaging
Source: PLoS One. 2020 Nov 16;15(11):e0242452. doi: 10.1371/journal.pone.0242452 (PMC7669278; doi:10.1371/journal.pone.0242452)
Supplement: S1 File — (PDF) [file pone.0242452.s003.pdf]

Supplementary: Probabilistic Optically-Selective Single-molecule Imaging Based Localization Encoded (POSSIBLE) Multiresolution Microscopy for Ultra-superresolution Imaging

Author : Partha Pratim Mondal

The List :

Supplementary 1: Cluster Analysis for SMLM and multiresolution POSSIBLE Microscopy

Supplementary 2: Cluster Area Analysis

Supplementary 3: Molecule Packing

Supplementary 4: Cluster Spread Density

Supplementary 5: Pairwise HA Distance Analysis

Supplementary 1: Cluster Analysis for SMLM and multiresolution POSSIBLE Microscopy

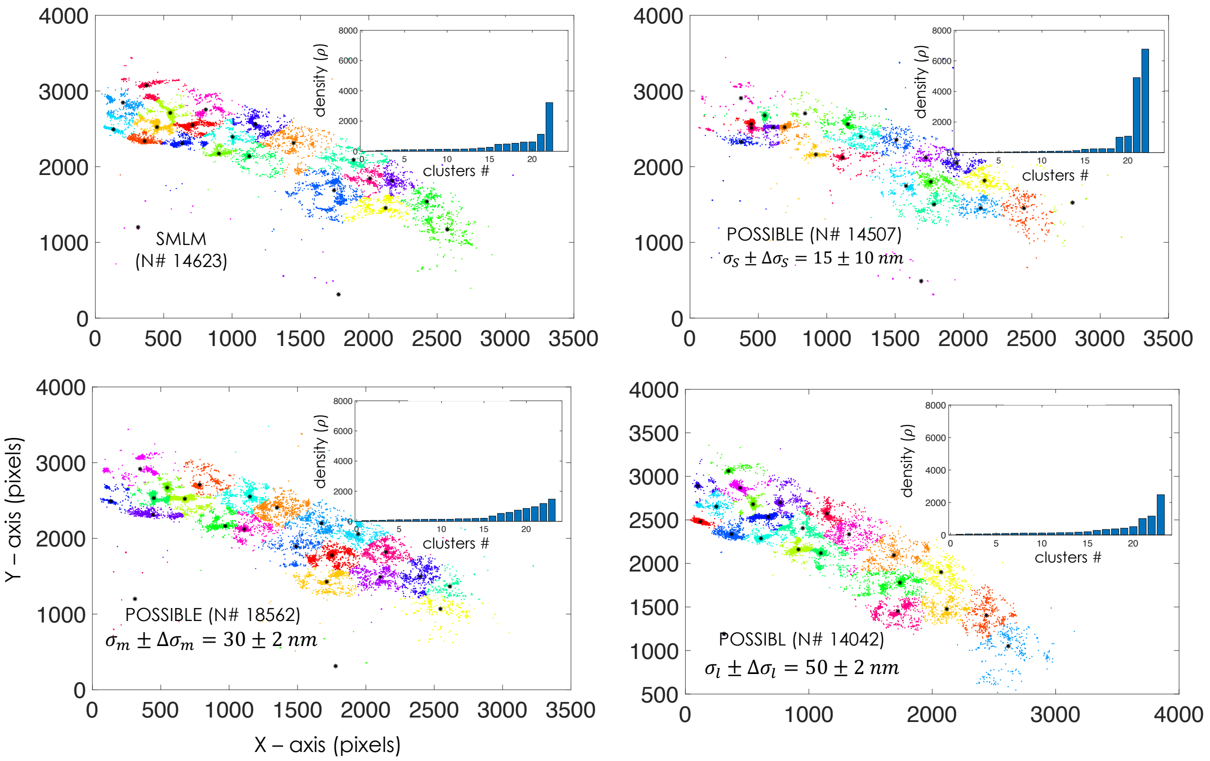

S1 Fig. K-means cluster analysis for multiresolution POSSIBLE microscopy. Three different classes are considered: highly-resolved clusters $G(\sigma_S,\ \Delta\sigma_S)$, moderately-resolved clusters $G(\sigma_m, \Delta\sigma_m)$ and poorly-resolved clusters $G(\sigma_l, \Delta\sigma_l)$.

Multiresolution clustering (at 3 different resolutions) enables better analysis of HA clusters in transfected NIH3T3 cells. Since K-means clustering algorithm requires user-defined input parameters such as the number of clusters in the superresolution map, one can define the # clusters by visual inspection. This also minimizes inaccurate clustering and in fact allows better clustering analysis resulting in an accurate extraction of relevant biological parameters such as, cluster area, molecule packing per cluster and cluster-spread density.

S1 Fig shows clustering for 4 different sub-datasets: SMLM (14623 HA molecules) and POSSIBLE. Three different resolution were chosen by specifying respective Gaussian distribution functions: $(\boldsymbol{G(\sigma_S = 15\,nm,\ \Delta\sigma_S = 10\,nm)},\ \boldsymbol{G(\sigma_S = 30\,nm,\ \Delta\sigma_S = 2\,nm)}\ ,\ \boldsymbol{G(\sigma_S = 50\,nm,\ \Delta\sigma_S = 2\,nm)})$. The

classification is essentially based on the size of PSF and size-bandwidth. Here, the bandwidth is chosen such that all the reconstructed maps have nearly same number of single molecules to enable comparison.

The inset in S1 Fig indicates the presence of highly dense clusters for POSSIBLE ($\boldsymbol{G}(\boldsymbol{\sigma_S}, \boldsymbol{\Delta\sigma_S})$). On the other hand, dense clusters are missing at low resolution ($\boldsymbol{G}(\boldsymbol{\sigma_l}, \boldsymbol{\Delta\sigma_l})$) resembling SMLM. This is predominantly due to nearly same mean PSF size for SMLM (size ≈ 40 nm) and POSSIBLE-$\boldsymbol{G}(\boldsymbol{\sigma_l}, \boldsymbol{\Delta\sigma_l})$ (size ≈ 15 nm). Specifically, nearly 10% of the clusters are of high density as revealed by $\boldsymbol{G}(\boldsymbol{\sigma_S} = \mathbf{15}\ \boldsymbol{nm}, \boldsymbol{\Delta\sigma_S} = \mathbf{10}\ \boldsymbol{nm})$**.** This is due to better resolving ability of POSSIBLE microscopy that shows the presence of sub-clusters within large clusters. The POSSIBLE microscopy is able to reconstruct super-resolution map using fortunate molecules with small average PSFs (σ = 15 nm) and a narrow Gaussian (variance, $\Delta\sigma$ = 10 nm) as compared to traditional SMLM (average PSF size = 40 and variance > 60 nm).

Supplementary 2: Cluster Area Analysis

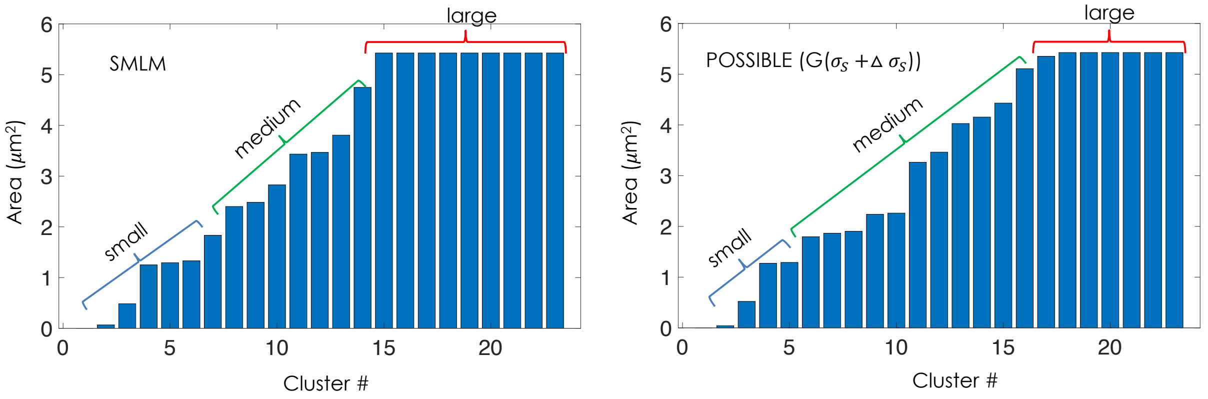

S2 Fig. Cluster area analysis for SMLM and POSSIBLE ($G(\sigma_S, \Delta\sigma_S)$) microscopy. The clusters are categorized into 3 major groups: Small, Medium and Large Clusters.

Cluster area is an important indicator of rate of infection. From S2 Fig, it is clear that the clusters range from 0.1 μm^2 to 6 μm^2. For convenience, we have broadly divided the clusters into 3 major groups: Small (0.1 - 2 μm^2), Medium (2 - 5 μm^2) and Large (5 - 6 μm^2). SMLM shows that majority of clusters are large in size, whereas POSSIBLE microscopy ($\boldsymbol{G(\sigma_S = 15\ nm, \Delta\sigma_S = 10\ nm)}$.) reveals that most of the clusters are in the medium range and relatively large clusters are less. This indicates that the large clusters in SMLM are indeed composed of relatively small sub-clusters as revealed by POSIBLE microscopy and traditional SMLM is unable to resolved large clusters. The advantage of POSSIBLE over SMLM is quite evident.

Supplementary 3: Molecule Packing (Number of HA molecules per cluster)

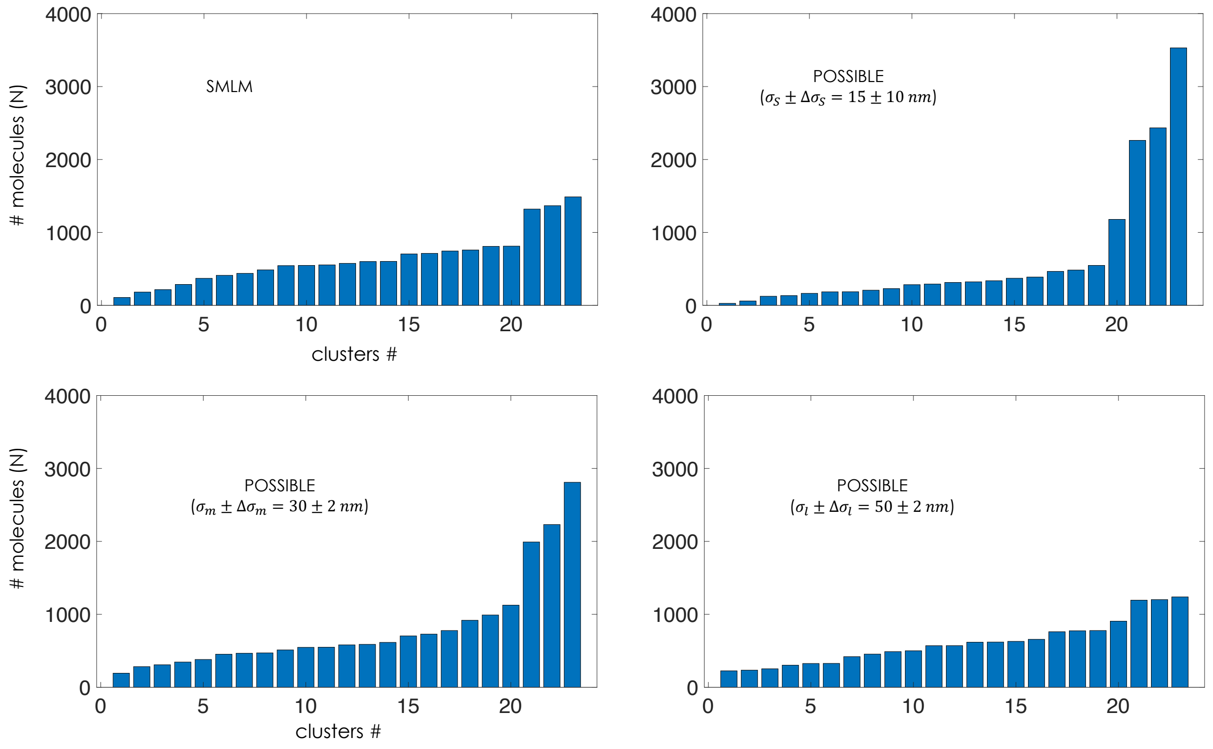

S3 Fig. Molecule packing analysis for SMLM and POSSIBLE ($G(\sigma_S, \Delta\sigma_S)$, $G(\sigma_m, \Delta\sigma_m)$ and $G(\sigma_l, \Delta\sigma_l)$) microscopy.

The number of HA molecules in the clusters is critical to virion maturation. S3 Fig shows the number of molecules in the clusters for SMLM and POSSIBLE ($\boldsymbol{G(\sigma_S, \Delta\sigma_S)}$, $\boldsymbol{G(\sigma_m, \Delta\sigma_m)}$ and $\boldsymbol{G(\sigma_l, \Delta\sigma_l)}$) microscopy. One can immediately notice that POSSIBLE ($\boldsymbol{G(\sigma_S, \Delta\sigma_S)}$ and $\boldsymbol{G(\sigma_m, \Delta\sigma_m)}$) microscopy reveals a good fraction of clusters with large HA molecules (> 2000) , whereas SMLM shows that most of the clusters have < 1200 molecules. So POSSIBLE microscopy indicates the presence of abnormally-high HA molecules in some clusters that are missed by SMLM.

Supplementary 4: Cluster Spread Density Analysis

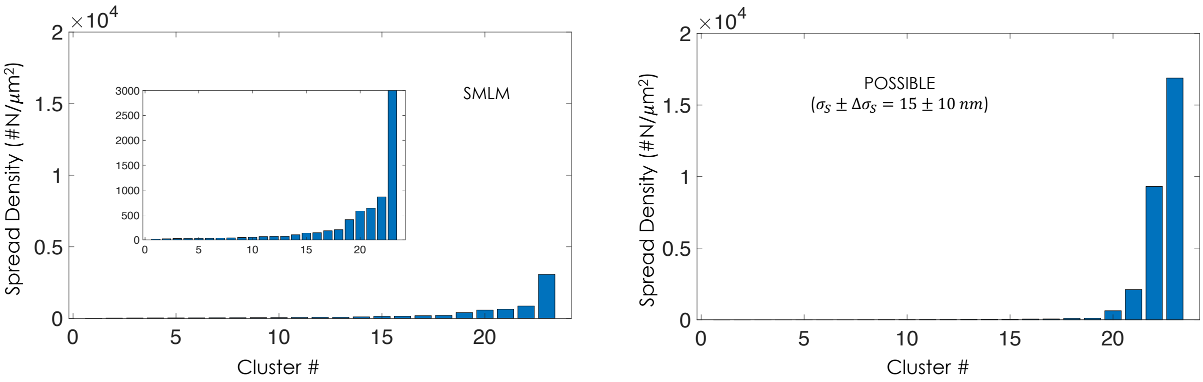

S4 Fig. Cluster spread-density analysis for SMLM and POSSIBLE ($G(\sigma_S, \Delta\sigma_S)$) microscopy.

An important aspect of HA clustering during influenza infection is the cluster spread-density which is the ratio of number of HA molecules in a cluster and the cluster spread-area.

The average cluster-spread radial-distance (measured in terms of square of Euclidean distances within a cluster) is defined as,

$$r_i = \frac{1}{N_i}\sum_{j=1}^{N_i}\left[(x_j - \beta_i)^2 + (y_j - \gamma_i)^2\right]^{1/2} \quad (1)$$

where, $\{x_j, y_j\}$ are the position coordinate of molecule ***j*** in the cluster ***i***, and $\{\beta_i, \gamma_i\}$ is the centroid. ***N****i* is the total number of molecules contained in the cluster ***i***.

The spread-area is defined as,

$$A_i = \pi\, r_i^2 \quad (2)$$

Finally, the cluster-spread density can be calculated as the ratio of number of molecules in a cluster ***i*** and cluster spread-area,

$$\rho_i = N_i \,/\, A_i \quad (3)$$

The spread-density for POSSIBLE reveal that it is way larger than that for traditional SMLM. Thus, spread-density is a highly sensitive parameter that other for determining dense

clusters. The inset shows that the maximum spread density for cluster in SMLM of about 3000 / μm^2, whereas the value is >6-fold for POSSIBLE. We call these abnormal clusters as, super-clusters. This is possible due to better representation of super-resolved image using small-sized PSFs (of fortunate molecules), thereby revealing the true nature of spread-density during infection.

Supplementary 5: Pairwise HA Distance Analysis

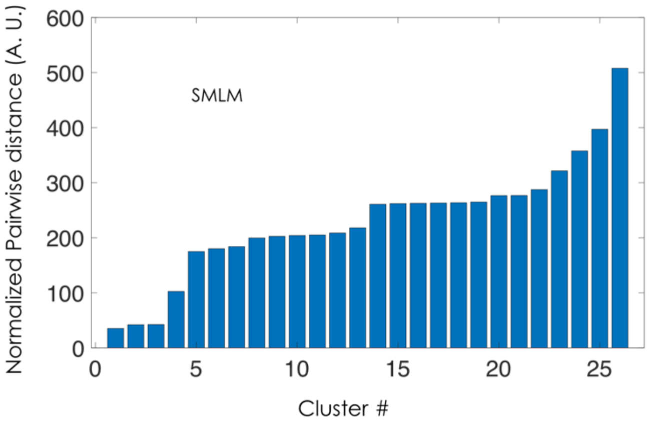

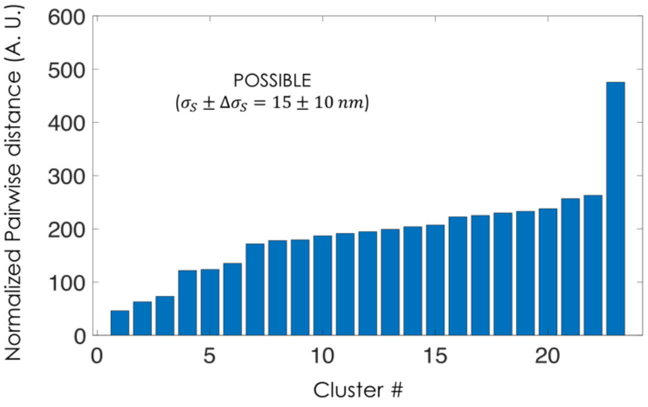

S5 Fig . HA pairwise-distance analysis for SMLM and POSSIBLE ($G(\sigma_S, \Delta\sigma_S)$) microscopy.

The pairwise HA molecule distance is a good indicator of cluster-spread. A large value indicates the distance over which the cluster is spread and vice-versa. The pairwise-distance between the molecules located at p(x,y) and q(x',y') is based on Euclidean distance,

$$d_j = \sqrt{(x_j - x_j')^2 + (y_j - y_j')^2}$$

and, the sum of pairwise distances is given by,

$$d = \sum_{j=1}^{n_i} d_j$$

where, n_i is the number of molecules in the cluster C_i.

S5 Fig shows the pairwise HA-HA single molecule distances within the clusters for both SMLM and POSSIBLE microscopy. The histogram indicates that majority of clusters have small *d*-value for POSSIBLE when compared to SMLM. This indicates an enhanced HA-HA interactions within the cluster and as a consequence small-area spread for POSSIBLE microscopy, whereas SMLM indicates clusters spreading over large area. This is in consistence with the observation that clusters are highly concentrated and localized post 24 Hrs of transfection [1][2]. So, POSSIBLE microscopy is able to precisely determine cluster-spread during influenza infection.

References:

1. Nikki M. Curthoys, Michael J.Mlodzianoski, Matthew Parent, Michael B.Butler, Prakash Raut, Jaqulin Wallace, Jennifer Lilieholm, Kashif Mehmood1, Melissa S. Maginnis, Hang Waters, Brad Busse, Joshua Zimmerberg, Samuel T.Hess, Influenza Hemagglutinin Modulates Phosphatidylinositol 4,5-Bisphosphate Membrane Clustering, Biophys. Jl., 116 893-909 (2019).

2. Parton DL, Tek A, Baaden M, Sansom MSP (2013) Formation of Raft-Like Assemblies within Clusters of Influenza Hemagglutinin Observed by MD Simulations. PLoS Comput Biol 9, e1003034 (2013).

Supplementary Videos

Supplementary Video 1: Raw images (first 100 frames) of single molecules recorded at 30 Hz and with an EMCCD camera with a gain of 250. On an average, 17.3 single molecules were observed per frame.

Supplementary Video 2: Raw data (first 100 frames) showing the extraction of fortunate molecules from the recorded single molecule data after Gaussian filtering. An average of 1.45 single molecules were observed per frame.
